# The highs and lows of monoamine oxidase as molecular target in cancer: an updated review

**DOI:** 10.1007/s11010-024-05192-w

**Published:** 2024-12-23

**Authors:** Iasmina M. Hâncu, Silvia Giuchici, Adina V. Furdui-Lința, Bogdan Lolescu, Adrian Sturza, Danina M. Muntean, Maria D. Dănilă, Rodica Lighezan

**Affiliations:** 1 Doctoral School of Medicine, “Victor Babeș” University of Medicine and Pharmacy of Timișoara, Timișoara, Romania; 2 Department III Functional Sciences-Pathophysiology, “Victor Babeș” University of Medicine and Pharmacy of Timișoara, Eftimie Murgu Sq., no.2, 300041 Timișoara, Romania; 3 Centre for Translational Research and Systems Medicine, “Victor Babeș” University of Medicine and Pharmacy of Timișoara, Timișoara, Romania; 4 Department XIII Infectious Diseases-Parasitology, “Victor Babeș” University of Medicine and Pharmacy of Timișoara, Timișoara, Romania

**Keywords:** Monoamine oxidase (MAO), Cancer, MAO inhibitors, Drug repurposing

## Abstract

The global burden of cancer as a major cause of death and invalidity has been constantly increasing in the past decades. Monoamine oxidases (MAO) with two isoforms, MAO-A and MAO-B, are mammalian mitochondrial enzymes responsible for the oxidative deamination of neurotransmitters and amines in the central nervous system and peripheral tissues with the constant generation of hydrogen peroxide as the main deleterious ancillary product. However, given the complexity of cancer biology, MAO involvement in tumorigenesis is multifaceted with different tumors displaying either an increased or decreased MAO profile. MAO inhibitors are currently approved for the treatment of neurodegenerative diseases (mainly, Parkinson’s disease) and as secondary/adjunctive therapeutic options for the treatment of major depression. Herein, we review the literature characterizing MAO’s involvement and the putative role of MAO inhibitors in several malignancies, and also provide perspectives regarding the potential biomarker role that MAO could play in the future in oncology.

**Supplementary Information:**

The online version contains supplementary material available at 10.1007/s11010-024-05192-w.

## Introduction

To date, cancer remains one of the most feared adversaries for modern medicine. The latest global cancer statistics showed an alarming increase in the disease incidence; the estimates are that approximately 1 in 5 people worldwide will develop a malignancy during their lifetime and by 2050 the number of diagnosed cases will have escalated by 77% [1].

The demand for efficient cancer therapies is urgent but with an estimated requirement of 10–15 years and up to US$ 2 billion investment for the clinical approval of any new drug, the translation from therapeutic possibility to reality is severely delayed [2]. To overcome this issue, drug repurposing has emerged as a viable alternative whereby existing, clinically used, non-oncological drugs with a well-documented pharmacokinetic and safety profile can be repositioned as anticancer medication. Recent advances regarding computational methods and drug screening technologies have identified multiple drug classes for oncological repurposing [3], including monoamine oxidase (MAOI) inhibitors [4].

Genes located on the short arm of the X chromosome encode for the expression of the monoamine oxidase (MAO), a flavoenzyme with 2 isoforms, MAO-A and MAO-B [5].

Monoamine oxidases are mammalian mitochondrial enzymes responsible for the oxidative deamination of neurotransmitters and endogenous and dietary amines in the central nervous system and peripheral tissues, with the constant generation of hydrogen peroxide as the main deleterious ancillary product. MAO-A is involved in the oxidative deamination of serotonin, epinephrine, norepinephrine, and dopamine, while MAO-B preferentially catalyzes the conversion of 2-phenylethylamine, benzylamine, and dopamine [6].

Besides their well-known role in the pathogenesis of neurodegenerative diseases and depressive disorders [7], the contribution of MAO-related oxidative stress to cardiovascular and metabolic diseases in both animal models and humans has been only more recently demonstrated [8–11]. Similarly, a substantial body of evidence has consistently linked, mainly in the past decades, the modified MAO activity and/or expression to various types of cancer [12]. Of note, Wattenberg reported more than sixty years ago that colon carcinoma cells display a decreased MAO activity, and this pattern is especially present in poorly differentiated elements [13]. However, the role of MAO in cancer pathogenesis is far from being elucidated, as many studies have produced conflicting results. The MAO-dependent metabolism of the above-mentioned neurotransmitters constantly yields H_2_O_2_ and ammonia. These compounds can reach toxic levels in case of increased MAO tissue expression or activity, potentially leading to mitochondrial toxicity, DNA damage, and ultimately tumorigenesis [14]. Consequently, the repurposing of MAO inhibitors for the oncology field has yielded promising results across multiple cancer types including glioma [15], prostate cancer [16], lung cancer [17], colorectal cancer [18], or breast cancer [14]. Moreover, MAO-A has recently emerged as a possible immune checkpoint that modulates tumor-associated immune cell metabolism, thereby influencing immunotherapy efficacy [19]. However, downregulation of MAO has also been described as tumorigenic in several tissues, most likely due to the resulting buildup of serotonin, dopamine, epinephrine, and norepinephrine which interact with their specific receptors and can have cancer-promoting effects [20–22]. MAO seemingly plays a double-edged role in oncogenesis with either a tumor-promoting or tumor-suppressive phenotype depending on cancer type-specific mechanisms including gene expression modifications leading to its up- or downregulation [23].

Noteworthy, depression not only impacts 20% of people globally [24] but among cancer patients its prevalence is four times higher than in the general population [25]. Thus, the therapeutic repurposing of MAO inhibitors for certain malignancies would provide a multi-target approach with the dual advantage of antidepressant as well as antitumor effects. Conversely, in cancers where MAO downregulation is a proven contributor to tumorigenesis, the use of MAO inhibitors as antidepressants should be reconsidered due to the possible exacerbation of the primary malignant condition.

The aim of the present review was to summarize the literature data regarding the aberrant MAO expression in various tumors, tackling both its increased and decreased expression, as well as the putative beneficial role of MAO inhibitors in the former case (alone or in combined administration with chemotherapeutics). Lastly, we briefly outlined the potential of MAO expression assessment as a cancer biomarker.

## MAO up-regulation in tumors and the benefits of MAO inhibitors

MAO overexpression is a prominent common denominator involved in the development and progression of various neoplastic conditions, hence the success that stand-alone or add-on MAO-inhibitor-based therapies have registered in several preclinical and a couple of clinical settings. MAO inhibitors are classics in chemical neuroscience that still possess contemporary relevance. They were the first antidepressants in clinical use and are currently successfully marketed for the treatment of Parkinson’s and Alzheimer’s diseases [26]. Currently, MAO inhibitors are second-line antidepressants due to the introduction of selective serotonin reuptake inhibitors (SSRI) which generally have fewer side effects and food–drug interactions [26]. However, the recently published prescriber’s guide concerning MAO inhibitor administration for depression describes this treatment as potentially life-saving and safer than ever, stating that enduring misinformation unduly amplifies their perceived risk profile [27]. Nowadays, it is clear that the main adverse effects of MAO inhibition are largely avoidable: serotonin toxicity only occurs after co-administration with SSRI, while the tyramine pressor response is infrequent due to the greatly diminished tyramine content in contemporary foods [28]. This repositioning regarding the safety of MAO inhibitors has revived the interest of the scientific community and has encouraged a surge of drug repurposing studies in pathologies such as obesity, diabetes, rheumatoid arthritis, or cancer [4, 29, 30].

### Prostate cancer

MAOs are critical for the regulation of prostate basal progenitor cells. MAO-A/B ablation in mice resulted in prostate atrophy and decreased epithelial cell proliferation without any impact on apoptosis. In human prostate epithelial cells, both the silencing as well as the pharmacological inhibition of MAO-A/B mitigated prostate stemness. Conversely, hyperplasic prostate tissue displays an increased MAO-A expression at epithelial level, while the stroma compartment presents enhanced MAO-A and -B expression compared to normal prostates [31].

A wealth of literature has linked MAO-A to prostate cancer growth, metastasis, and therapy resistance via numerous mechanisms including oxidative stress, hypoxia, epithelial-to-mesenchymal transition, and modulation of the tumor microenvironment [32]. The pathogenic mechanisms that lead to high MAO-A expression in prostate cancer are quite complex. Activated androgen signaling and dysfunctional oncogenic signaling manifested by c-Myc activation and loss of phosphatase and tensin homolog (PTEN) and p53 onco-suppressors seem to act in concert to upregulate MAO-A at molecular level. These mechanisms, with particular emphasis on the androgen connection, might explain the differences in MAO expression and activated downstream mechanisms between prostate cancer and other malignancies [33]. Conversely, patients who display low MAO-A expression through a polymorphism of the MAO-A promoter have a reduced risk of prostate cancer development [34]. In xenograft murine prostate cancer models obtained using various human prostate cell lines or mouse MPC3 cells harboring double loss of the PTEN and p53 tumor suppressors, knockdown of MAO-A mitigated tumor cell proliferation [33]. These results were recapitulated in a prostate-specific PTEN knockout mouse model in which supplementary knockdown of MAO-A in prostate epithelial cells reduced the tumor size, invasiveness, and stemness. The PTEN knockout murine model mimics human prostate cancer as PTEN-null mice spontaneously develop prostatic hyperplasia which eventually turns into adenocarcinoma by 3–6 months of age [35].

True et al. were the first to connect high levels of MAO-A expression in prostate cancer epithelial cells with poorly differentiated morphological features (Gleason scores over 4) [36]. These results were confirmed in a subsequent study which additionally verified the expression of CD44, a marker of basal prostatic epithelial cells, and found it to have a concurrent expression with MAO-A, suggesting that high-grade prostate cancer may display a basal or perhaps a progenitor cell-like phenotype. Moreover, MAO-A expression was also positively associated with the preoperative serum PSA (prostate-specific antigen) concentration [37]. The same group proved that MAO-A inhibits the differentiation of basal epithelial cells into secretory cells, while clorgyline, the irreversible MAO-A inhibitor, counteracted this de-differentiated phenotype and induced the expression of the androgen receptor which in turn downregulated MAO-A expression in secretory-like cells [38]. In high-grade prostate cancer, clorgyline has both pro-differentiating as well as anti-oncogenic properties. It decreases the expression of EZH2, a protein involved in repressing genes necessary for differentiation, and counteracts pathways regulated by key oncogenes such as beta-catenin or ERBB2 [39]. Using metastatic castration-resistant prostate cancer VCaP cells that display high MAO-A expression, Flamand et al. recapitulated these beneficial results through in vitro as well as in vivo experiments. Thus, clorgyline treatment inhibited VCaP cell proliferation and altered their transcription pattern, while in a murine VCaP xenograft model, clorgyline delayed tumor growth [40].

The MAO-A-dependent accumulation of reactive oxygen species (ROS) in prostate cancer cells stabilizes hypoxia-inducible factor 1-alpha (HIF1α), which in turn activates vascular endothelial growth factor (VEGF) and its neuropilin-1 receptor (NRP1) leading to stimulation of the Akt/FOXO1 pathway. As a result, the transcription of TWIST1 is initiated and epithelial-to-mesenchymal transition (EMT) is induced [33]. EMT is characterized by the simultaneous loss of cell adhesion and enhanced cell mobility, leading to the invasive, migratory, and metastatic potential of cancer cells. Generally, tumors with a high degree of malignancy display increased levels of HIF1α, VEGF, and TWIST1, prompting Sblano et al. to suggest that together with the expression of MAO-A, these markers should be used to differentiate high-aggression from low-aggression tumors [4]. MAO-A knockdown in different prostate cancer cell lines counteracted the above-mentioned mechanisms and significantly attenuated EMT in vitro*,* while in prostate cancer xenograft mouse models, both MAO-A knockdown as well as inhibition through clorgyline reduced tumor growth [33]. These results are consistent with the poor prognosis and worse clinical outcomes that have been described in the case of prostate cancer patients with MAO-A-overexpressing tumors [33]. Survivin, an inhibitor of apoptosis, similarly impacts patient prognosis. In clinical prostate cancer tissues, both survivin as well as MAO-A levels are increased. The functionality of these two proteins is linked as survivin upregulates MAO-A expression during prostate cancer development, while MAO-A knockdown diminished survivin expression. Concomitant administration of survivin suppressants and MAO-A inhibitors significantly mitigated tumor cell growth and migration and counteracted cancer metastasis by decreasing the level of the extracellular matrix digestion enzyme MMP-9 [41].

Wu et al. observed that different prostate cancer cell lines obtained from bone metastases in murine intracardiac xenograft models display higher levels of MAO-A mRNA as compared to their parental counterparts in the primary tumor. Moreover, intracardiac injection of MAO-A-overexpressing PC3 prostate cancer cells resulted in accelerated bone metastasis and increased tumor burden versus PC3 cells with low MAO-A expression. Via a TWIST1-mediated mechanism, MAO-A activates Sonic hedgehog (Shh) signaling in malignant prostate cells, initiating a cross-talk with the surrounding osteoblasts to promote growth advantages for tumor cells in the bone microenvironment. Subsequently, osteoblasts release interleukin 6 (IL-6) and RANKL (receptor activator of nuclear factor kB ligand) which stimulate osteoclastogenesis with ensuing bone resorption. Disrupting the Shh-IL-6-RANKL signaling via clorgyline limited metastasis, decreased the number of osteoclasts, and prolonged mouse survival [42]. In a TWIST1-dependent manner, MAO-A also promotes tumor perineural invasion and facilitates tumor innervation during prostate cancer development. The pathogenic cascade comprises the activation of semaphorin 3C, a regulator of axon directional migration, with consequent cMET stimulation. Both in vitro perineural invasion of prostate cancer cells as well as tumor-infiltrating nerve fiber density and tumor growth and progression in mouse xenograft prostate cancer models were significantly reduced by clorgyline treatment [43]. Recently, Pc-MLB, a MAO-A-directed phthalocyanine photosensitizer was constructed. These compounds act by transforming intracellular oxygen molecules into highly cytotoxic ROS upon exposure to the matched light irradiation. Pc-MLB specifically targeted prostate cancer cells that overexpress MAO-A and reduced their proliferative, migratory, and metastatic potential in vitro [44].

The androgen receptor (AR) is pivotal for prostate cancer growth and progression. FOXA1 (Forkhead box protein A1) is a transcription factor which interacts with the AR to regulate gene expression. However, its mutation is a possible driver of tumor malignancy. In a recent in silico study, mining The Cancer Genome Atlas (TCGA) for transcriptomic data revealed 1525 differentially expressed genes between FOXA1 mutant and mutant-negative (control) prostate cancer groups. After matching this information with genes from the CancerMine database, the MAO-A gene appeared upregulated in the FOXA1 mutant group, supporting MAO-A inhibition in this high-risk population as an adjunctive or stand-alone therapy aimed at improving patient outcome [45].

Androgen deprivation therapy is the mainstay treatment for prostate cancer. However, most patients relapse after therapy escape and develop castration-resistant prostate cancer (CRPC) with reactivation of the AR despite the low androgen environment [32]. Although overall survival in this advanced form of malignancy has been greatly improved by the introduction of enzalutamide or abiraterone, approximately, 20% of patients treated with these second-generation androgen pathway inhibitors (API) progress toward highly aggressive neuroendocrine prostate cancer (NEPC) [46]. MAO-A is differentially expressed in prostate tumoral tissue according to the stage of the disease. In LNCaP prostate cancer cells that responded to castration-induced androgen deprivation, MAO-A mRNA expression decreased but was partially recovered upon progression to the castration-resistant phenotype [47]. A later study revealed that under low androgen conditions, AR promotes MAO-A transcription through binding to the gene promoter, thus facilitating androgen-independent growth of the LNCaP androgen-dependent cells [48]. In human CRPC samples, the increased expression of MAO-A exhibits a positive correlation with the AR. Wei et al. delved deeper into the interaction between AR and MAO-A and found that it is bidirectional and manifested both in androgen-responsive as well as CRPC cells. Specifically, AR binds to an intronic element in the MAO-A gene which in turn enables AR transcription via ROS/TWIST1-induced activation of Shh signaling leading to the upregulation of the AR co-activator YAP1. Silencing MAO-A has growth-suppressing effects in mouse prostate tumors, including the CRPC phenotype, while the anti-tumoral effect of enzalutamide, darolutamide, and apalutamide was greatly enhanced by MAO-A genetic or pharmacological inactivation in both androgen-dependent and CRPC cells [49]. These results are in agreement with a previous study demonstrating that clorgyline decreases the expression of the AR along with that of its target gene PSA and of AR-V7, the constitutively active splicing variant of AR that confers antiandrogen therapy resistance. Moreover, MAO-A inhibition had an additive effect to the anti-proliferative capacity of enzalutamide in androgen-responsive and irresponsive cells and restored sensitivity of enzalutamide-resistant cells to this API [50]. Enzalutamide administration enhances the expression of AR-V7 which binds to a response element on the MAO-A promoter region and transcriptionally upregulates its expression and increases its stability. This leads to HIF1α-induced enzalutamide resistance which can be delayed or reverted by clorgyline, the nonselective MAO inhibitor phenelzine or MAO-A silencing, further limiting tumor growth in in vivo models [16]. Acquired antiandrogen therapy resistance seems to depend on the glucocorticoid receptor (GR) as well. Prostate cancer cells can evade AR blockade-mediated cell death during treatment via elevated GR signaling, responsible for MAO-A upregulation in epithelial and stromal cells. Clorgyline coupled with either second-generation API or chemotherapeutics mitigated the proliferation and viability of androgen-dependent or -independent cells and long-term anti-androgen-treated cells [51]. In the context of CRPC, modified characteristics can also be detected in patient circulating tumor cells (CTC) and peripheral blood mononuclear cells (PBMC). MAO-A as well as AR-V7 expressions were upregulated in CTCs isolated from patients treated with enzalutamide [16], while MAO-A expression in patient PBMCs was positively correlated with bone metastasis, castration resistance, and the level of serum PSA [52].

The vast majority of NEPC cases are a result of the progression of API-resistant CRPC. In this particular state, prostate cancer cells are very difficult to destroy as they display lineage plasticity and acquire neuroendocrine traits characterized by the presence of neuroendocrine markers and limited dependence on AR signaling [53]. Downregulation of REST (repressor element-1 silencing transcription factor) is pivotal for NEPC induction. Lin et al. were the first to show that in neuroendocrine-differentiated prostate cancer cells, a reduced expression of REST promotes MAO-A upregulation which in turn mitigates androgen deprivation-induced apoptosis and activates autophagy and mitophagy in a ROS-dependent manner [54]. In a NEPC cell model obtained after long-term enzalutamide exposure, MAO-A expression was described as significantly increased and directly involved in neuroendocrine differentiation via mTOR/HIF1α signaling. Co-administration of clorgyline and enzalutamide reduced tumor growth and weight and delayed tumor progression in a mouse xenograft model of NEPC [55]. Recently, MAO-A-induced TWIST1 activation was reported to upregulate the expression of BRN2, a master neural transcription factor that prompts neuroendocrine differentiation in CRPC [56].

Advanced prostate adenocarcinoma is particularly resistant to the action of commonly used chemotherapeutic agents such as docetaxel [46]. Gordon et al. explored the mechanisms behind this drug resistance in relapsed patients and found that chemotherapy augmented MAO-A gene expression which further promoted malignant cell survival in a ROS/HIF1α-dependent manner. Inhibition of MAO-A via clorgyline administration enhanced docetaxel toxicity on prostate cancer cells [57].

Given the preclinical success in applying MAO-A inhibitors to treat prostate cancer, natural products have also been considered as alternative therapeutic candidates. In prostate cancer cells, curcumin inhibited MAO-A/mTOR/HIF1α signaling, thus suppressing epithelial-mesenchymal transition [58], while a synthesized flavonoid derivative with MAO-A inhibiting properties displayed anti-proliferative and anti-metastatic activities [59]. Moreover, Quercetin, Apigenin, and Luteolin potently target and inhibit MAO-A, as described in a molecular docking study screening a multitude of phyto-chemicals derived from African medicinal plants [60].

The role of MAO-A in prostate cancer is very complex as enhancing its catalytic activity with consequent overproduction of ROS may have tumor-suppressing properties in the presence of deficient intracellular antioxidant mechanisms. Administration of the polyphenol-rich fraction of Bergenia ligulata (PFBL) promoted significant MAO-A-dependent oxidative stress in prostate cancer cells with concomitant Akt inhibition resulting in GSK-3β activation and suppression of the NRF2-derived antioxidant response. Consequently, the malignant cells underwent ROS-induced apoptotic death, while in a mouse xenograft model, PFBL decreased prostate tumor volume both alone as well as in co-administration with Paclitaxel [61].

As opposed to MAO-A, data regarding the impact of MAO-B on prostate cancer are scarce. Selegiline, the selective MAO-B inhibitor, was reported more than two decades ago to mitigate gamma radiation or cisplatin-induced apoptosis in nonmalignant human cells, without displaying any protective effect on prostate cancer and bladder carcinoma cells exposed to the same cancer treatment [62]. Recently, Huang et al. demonstrated that in various epithelial prostate cancer cell lines, the induced overexpression of MAO-B attenuated their proliferative, migratory, and invasive potential, while treatment with selegiline reversed these beneficial effects. Clinically, low MAO-B expression was associated with more aggressive tumor features and a poorer prognosis, while several MAO-B single-nucleotide polymorphisms were linked to greater prostate cancer metastasis capacity via decreased MAO-B enzymatic activity [63]. However, the interaction between MAO-A and -B during tumorigenesis might be equally important as the stand-alone effects of these isoforms since administration of pargyline, the MAO-A and -B irreversible inhibitor, decreased the proliferation and induced apoptosis of LNCaP-LN3 prostate cancer cells [64].

Most of the current research and therapeutic interventions in prostate cancer are focused on epithelial tumor cells. However, prostate cancer development is greatly influenced by stromal—epithelial cells cross-talk that supports the reprogramming of naïve stroma into a tumor-promoting phenotype. In a recently published study, Pu et al. demonstrated that MAO-A is the predominant MAO isoform in human prostate tumoral epithelial cells, while in stromal cells MAO-B is highly abundant. Importantly, the level of expression of these enzymes in their respective compartment increased in parallel with the Gleason score. Stromal MAO-B overexpression was associated with prostate cancer cell proliferation, migration, and invasion with a poor patient prognosis, while epithelial MAO-B level did not influence overall survival. Mechanistically, MAO-B-derived ROS induced a transition of the stroma toward a reactive tumorigenic phenotype with CXCL12 activation via TWIST1 synergy with TGFβ signaling. The CXCL12/CXCR4 paracrine signaling promoted stromal–epithelial interactions with resulting aggressive tumor behavior. In a mouse orthotopic xenograft, prostate cancer model treatment with selegiline decreased stromal expression of CXCL12 and restricted tumor growth [65]. Previously, the same group had shown that MAO-A upregulation in prostate stromal fibroblasts promotes tumorigenesis via ROS generation and TWIST1-mediated paracrine IL-6/STAT3 signaling, responsible for increasing CD44 in prostate cancer cells which points to the maintenance of a de-differentiated phenotype. The administration of a MAO-A inhibitor mitigated prostate tumor growth in mice [66]. Moreover, cancer-associated fibroblasts present in the reactive tumoral stroma can activate MAO-A in prostate cancer cells with consequent ROS overproduction and mTOR/HIF1α signaling that leads to EMT and the increased expression of the CXCR4 and IL-6 receptor, promoting a migratory and aggressive prostate tumor phenotype [58]. Noteworthy, TNF-α and IL-6 seem to stimulate MAO-B expression in normal human prostate stromal fibroblasts, pointing to a possible mechanistic link between MAO isoforms in the initial communication between tumor cells and normal stroma [65].

The wealth of literature data describing the efficacy of MAO inhibition in prostate cancer has prompted the first clinical trial that supports MAO inhibitor applicability in human cancer. Specifically, in a phase 2 clinical trial conducted on patients with biochemical recurrent castrate-sensitive prostate cancer, phenelzine elicited PSA decline in 55% of the study population in a safe and well-tolerated manner with most patients being able to reach the target dose. The adverse reactions were minimal and included dizziness and syncope [67]. Given the promising result, Jacobs et al. focused on identifying novel MAO inhibitors with a reduced blood–brain barrier penetration in order to limit neurological side effects. Recently, this group designed and synthesized a series of clorgyline-based compounds with a modified structure that retained the cytotoxic effect against prostate cancer cells but displayed a drastic decrease of central nervous system penetration [68].

### Other urological cancers

Although the mechanistic link between MAOs and prostate cancer has been extensively studied, bladder cancer research has only recently focused on the possible MAO involvement in tumorigenesis. Both MAO isoforms are present in the AY27 rat bladder cancer cell line and human urothelial tumor explants. Selective MAO-A and -B inhibition limited cell cycle progression and mitigated cancer cell proliferation in a ROS-dependent manner. However, only MAO-A inhibition was able to suppress cell motility. Moreover, MAO inhibitors impaired cancer cell glucose metabolism by decreasing the expression of glucose transporter 1 (GLUT1) and hexokinase 2 (HK2) with reduced glycolysis and oxidative phosphorylation in conjunction with pyruvate depletion [69]. A subsequent study confirmed the presence of MAO-A and -B in all stages and grades of non-muscle (NMIBC) as well as muscle invasive bladder cancer [70]. OncoTherad immunotherapy is a novel treatment option for patients with NMIBC that has become unresponsive to the standard Bacillus Calmette-Guérin immunotherapy. The promising OncoTherad results in bladder cancer treatment have prompted a comprehensive analysis of the intracellular mechanisms it triggers. A recent report has revealed that OncoTherad immunotherapy significantly decreased MAO-B immunoreactivities in tumor tissues collected from NMIBC patients who no longer responded to standard therapy [71].

Similarly to reports in prostate cancer, high-grade renal cell carcinomas were associated to elevated expression of MAO-A which may play an important role in maintaining a de-differentiated phenotype with consequent aggressive tumor behavior [72].

### Breast cancer

Breast cancer is a heterogeneous neoplasm with a multifaceted clinical, histological, molecular, and genetic presentation. According to tumoral expression of the estrogen receptor (ER), progesterone receptor (PR), and human epidermal growth factor receptor 2 (HER-2), breast cancer is classified into four molecular subtypes: luminal A, luminal B, HER-2 type, and triple-negative breast cancer (TNBC). The latter is diagnosed when ER, PR, and HER-2 are negative in immunohistochemical investigations. Noteworthy, aside from hormone receptors, MAO isoforms are differentially expressed in breast cancer subclasses. TNBC staining revealed elevated expression of MAO-B [73], while the MAO-A gene is mostly downregulated compared with normal ductal cells [74] which seems to have a negative impact on TNBC development. The Cancer Genome Atlas (TCGA) and Gluck breast cancer databases mining revealed that MAO-A downregulation generally occurred in invasive breast carcinomas, possibly as a survival technique employed by cancer cells to conserve amino acid resources necessary for protein synthesis and cell growth [75]. Luminal A and B subtypes display the highest levels of MAO-A, which in turn is associated with a low histological grade and ER and PR positivity. However, in HER-2 breast cancer, the presence of MAO-A was linked to short disease-free survival [73].

More than three decades ago in a chemically induced rat model of breast cancer, Lizcano et al. described an increase of MAO-A and a decrease of MAO-B activities in highly malignant tumors [76]. Working on the premise that increased catecholamine activity in the brain might prevent the development of mammary and pituitary tumors, Thyagarajan et al. demonstrated a reduced incidence of these neoplasms in acyclic female rats undergoing long-term treatment with deprenyl, the selective irreversible MAO-B inhibitor [77]. A few years later, L-deprenyl (selegiline) was reported to decrease tumor incidence and number in a carcinogen-induced rat mammary cancer model. The authors hypothesized that the described effect was in relation to reduced prolactin secretion and a possible protective action on tuberoinfundibular dopaminergic neurons in the medial basal hypothalamus associated with catecholamine metabolism inhibition [78, 79]. Subsequently, in a more detailed study, splenic noradrenergic innervation along with splenic levels of norepinephrine, IL-2, interferon γ (IFN-γ), and natural killer cell activity were described as reduced in mammary tumor-bearing rats versus healthy controls. Treatment with L-deprenyl reversed this cancer-associated decline and increased IL-2 and IFN-γ production in the draining lymph nodes. Therefore, the improvement of anti-tumoral immune responses and of sympathetic noradrenergic activity at splenic and lymph node level might account for the deprenyl-induced prevention of mammary tumor development. In vitro incubation of human TNBC and luminal A-type cells with deprenyl partly inhibited proliferation of ER + cells but had no effect on ER − cells [80]. Recently, selegiline proved a potent cytotoxic effect against TNBC and luminal A-type breast cancer cells through a ROS-independent mechanism with concomitant inhibition of protein kinase C phosphorylation [81]. In a luminal A-type breast cancer cell line pargyline halted the cell cycle in the G1 phase, thus inhibiting cellular growth and proliferation [82]. Recently, Alkhawaldeh and Bardaweel tested several clorgyline-based MAO-A inhibitors and showed that they promoted apoptosis and suppressed the proliferation, invasiveness, and colony-forming abilities of a luminal A and TNBC-type breast cancer cell line, respectively. Moreover, the Bcl-2 and VEGF gene expressions of tumoral cells were decreased compared to untreated controls. Co-administration of the MAO-A inhibitors with doxorubicin or raloxifene reduced the anticancer drug dose needed for a significant anti-proliferative effect [14]. However, in a previous study, Satram-Maharaj et al. emphasized the importance of the EMT status over the presence of the ER receptor in determining cellular malignant behavior. The comparative evaluation of MCF-7 luminal A-type cells and MDA-MB-231 TNBC-type cells revealed that the former display a significantly lower level of MAO-A catalytic activity versus the latter. This might account for the minor clorgyline-induced proliferation suppression described in the MCF-7 line. MDA-MB-231 are a model of post-EMT cells. MAO-A inhibition in this cell line produced a potent anti-proliferative response and a decreased colony-forming ability but was associated with an increment in cellular invasiveness and migratory capacity. This detrimental effect was due to mesenchymal-to-epithelial transition (MET) [83] which is the reverse process of EMT whereby tumor cells that have already migrated resume their epithelial characteristics in the metastatic location [84]. MET is correlated to advanced metastatic cancer stages; therefore, caution should be exercised when considering the administration of MAO inhibitors in these cases. A subsequent study seems to confirm heightened invasiveness of breast tumor cells in the presence of low MAO-A activity. TNBC cell lines treated with CoCl2 in order to mimic the hypoxic tumoral environment displayed an amplified IL-6/IL-6 receptor level responsible for sustained MAO-A inhibition which in turn promoted tumor angiogenesis and invasion. Conversely, genetic or pharmacological inhibition of IL-6/IL-6 receptor signaling upregulated MAO-A expression and suppressed these aggressive tumor aspects [85].

Breast tumor-initiating cells (BTIC) are a sporadic stem-like neoplastic population involved in cancer initiation, metastasis, and therapy resistance. In vitro investigation of BTIC is performed by growing breast tumor cell lines as tumorspheres since culturing them as adherent cells has proven to drastically decrease their frequency. Gwynne et al. demonstrated that tumorspheres obtained from cell lines representative of all breast cancer clinical subtypes exhibit an increased MAO-A expression which is associated with BTIC-related properties. MAO-A inhibition through clorgyline and especially through tetrindole induced a decline in tumorsphere-forming cells frequency. In a subsequent analysis of raw transcriptomic datasets originating from other studies, the authors noted that human breast cancer cell lines with acquired anticancer drug resistance share as a common feature the increased expression of MAO-A, which correlates with poor recurrence-free survival of patients diagnosed with the TNBC or HER-2^+^/ER^−^ subtypes [86].

Importantly, MAO is not only involved in breast cancer pathogenesis but also in the mental adaptation of patients to this condition. Women with early breast cancer carrying the genetic variable number of tandem repeat (VNTR) MAO-A polymorphism displaying decreased MAO-A functionality presented a less anxious preoccupation at follow-up sessions. Thus, blood or oral mucosa sampling for MAO-A activity assessment might represent a possible method to identify subjects in greater need of psychological support [87].

### Colon cancer

More than 60 years ago, a decreased MAO activity was described in colon carcinoma cells, especially in elements with poor differentiation, revealing the possibility that higher colon cancer malignancy is associated with low MAO activity [13]. These results seem to be consistent with a population-based case–control study with a follow-up period of 12 years that found an increased incidence of colorectal cancer among patients treated with MAO inhibitors. However, the small effect size of this correlation raises the possibility of a Type I error [88]. Mikula et al. evaluated the modification of different proteins in the transition from normal colonic mucosa to colon adenoma and adenocarcinoma and found that MAO-A expression gradually declined along malignant progression with the lowest levels being registered in adenocarcinoma samples [89]. In a more recent analysis, MAO-B expression was found to be highly elevated in conjunction with low levels of MAO-A in colorectal cancer tissues compared to their non-tumor counterparts. This increased expression of MAO-B was associated with a poor prognosis and higher recurrence rate. The analysis of the available gene expression levels in the TCGA colon cancer cohort demonstrated that MAO-B has a positive correlation with mesenchymal gene expressions and a negative connection with epithelial-related genes, thus revealing a possible role in EMT progression [90]. Importantly, MAO-A expression was not ubiquitously low across the entire study population but only in 62.1% of cases. The fact that some colorectal cancer samples exhibit high levels of MAO-A despite the low-expression trend might partly explain why in two subsequent studies employing different cell lines isolated from human colorectal cancer tissues MAO-A expression was reported to be increased. Indeed, Ayoup et al. evaluated the antineoplastic potential of novel dual MMP-9/MAO-A inhibitors and described a potent migratory-suppressing effect along with significant HIF1α downregulation in HCT116 human colorectal cancer cells that overexpress MMP-9 and MAO-A [91]. Recently, Bardaweel et al. applied a computational systems biology approach to prove the anticancer properties of MAO-A inhibition. Subsequently, these results were confirmed by experimental colorectal cancer cell line protocols that revealed anti-proliferative and antimigratory effects of clorgyline treatment as monotherapy or in co-administration with doxorubicin [18].

### Lung cancer

Lung neoplasms, divide into small cell lung cancer (SCLC) and non-small cell lung cancer (NSCLC), are the most prevalent cause of cancer-related mortality worldwide, with a well-documented MAO involvement in their pathogenesis. Compared to adjacent non-tumoral lung samples, NSCLC tissues display high expression of MAO-A protein and mRNA that correlates well with the late stage of NSCLC development and the existence of lymph node metastases. This is most likely due to MAO-A involvement in mediating EMT via increased expression of the mesenchymal marker N-cadherin and of the TWIST transcription factor and downregulation of the epithelial protein E-cadherin [92]. These results are consistent with a bioinformatics analysis study that revealed that the MAO-A gene is upregulated and differentially expressed in lung cancer tissue as opposed to para-carcinoma samples [93]. Moreover, mining TCGA and GEO (Gene Expression Omnibus) datasets for metabolism-related genes in the tumor microenvironment of lung adenocarcinoma revealed MAO-B expression as a protective factor for overall survival that was used together with other genes to successfully construct a prediction model for immune cell infiltration in lung adenocarcinoma [94].

The prognosis of lung cancer patients, especially those diagnosed with the NSCLC subtype, is poor despite the multitude of therapeutic strategies that can be employed [95]. NSCLC cells become radioresistant over time, and as a result of radiotherapy failure, most cases develop secondary lung cancer or distant metastasis which substantially increases mortality. Danshensu, a component of traditional oriental medicine, elicits radio-sensitization of NSCLC cells via inhibition of MAO-B which further decreases NF-κB (nuclear factor-κB) signaling with consequent suppression of EMT and downregulation of pro-survival and pro-inflammatory genes. Moreover, in a mouse xenograft model, danshensu increased lung tumor sensitization to ionizing radiation [96]. Paclitaxel is a traditional chemotherapeutic agent whose efficacy is frequently limited by drug resistance. In order to overcome this detrimental effect, the group of Wang D. repurposed G10 and G11, two MAO-A inhibitor-heptamethine carbocyanine dye conjugates, that had previously shown promise in mitigating prostate cancer cell growth [97]. NSCLC cells treated with paclitaxel for three months became unresponsive to this drug and exhibited high MAO-A expression levels. G10 and G11 mitigated the viability, migration, and invasiveness of paclitaxel-resistant cells by inhibiting Akt and HIF1α signaling with downregulation of metastasis-facilitating matrix metallopeptidases (MMP2 and MMP14) and VEGF. Moreover, in mouse xenograft NSCLC models, co-administration of G10 or G11 together with paclitaxel significantly inhibited tumor growth and metastasis, respectively [98, 99]. Since MAO inhibition has shown great lung tumor-suppressing potential, recently Bardaweel et al. designed and synthesized novel MAO-A inhibitors that reduced NSCLC cell lines’ proliferation in a dose- and time-dependent manner [17].

The signals leading to modified tumoral expression of MAO are varied and important to identify. Dhabal et al. demonstrated that interleukin 13 (IL-13) induces MAO-A expression with consequent MAO-A-dependent oxidative stress and cellular migration in primary human monocytes and a NSCLC cell line via mechanisms that depend on the STAT6 and PPARγ transcription factors and 15-lipoxygenase [100]. Human papillomavirus (HPV) with its associated HPV-16 E7 oncoprotein was reported to induce EMT and HIF1α protein accumulation in NSCLC cell lines via the stimulation of MAO-A expression. MAO-A knockout inhibited HPV-16 E7-induced cellular oxidative stress and metastatic capacity in vitro, while in vivo it mitigated HPV-16 E7-dependent tumor growth and metastasis along with the expression of EMT protein markers and HIF1α [101]. A recent biostatistical analysis study performed on TCGA datasets concerning AMPK (AMP-activated protein kinase) expression patterns in lung cancer, adenocarcinoma, and squamous cell carcinoma, respectively, revealed that AMPKα1 was poorly expressed across all pulmonary neoplasm subtypes, which in turn correlated with a high metastatic potential and a decreased overall survival rate. The authors expanded on this by performing cell culture experiments and demonstrated that AMPK activators mitigate NSCLC and colon cancer cell lines’ proliferation and migration via suppression of p38 mitogen-activated protein kinases (p38MAPK) activity with downregulation of MAO-A expression and activity [102].

Interestingly, dietary short chain (caproic and decanoic) and long chain (oleic and stearic) fatty acids were reported to stimulate MAO-A induction in A549 lung cancer cells. Consequently, treatment of lung carcinoma cells with oleic and stearic acid increased ROS generation in the presence of tyramine and further mitigated cell proliferation [103].

### Neuroblastoma

Neuroblastoma originates from undifferentiated neural crest cells and represents the most frequent childhood extracranial solid tumor [104]. In SH-SY5Y human neuroblastoma cells, Fitzgerald et al. demonstrated that staurosporine-induced apoptosis is mediated by an amplification in MAO-A activity and oxidative stress, while MAO-A inhibition mitigated apoptotic signaling [105]. In a model of MAO-A-overexpressing neuroblastoma cells, the increment in ROS levels resulted in autophagy via Bcl-2 phosphorylation and mitophagy with maintained cellular ATP concentrations and increased complex IV activity/protein levels without complete mitochondrial depolarization. However, substrate availability might be the determining factor of cell fate as addition of increasing doses of tyramine caused significant cell death in MAO-A overexpressing cells, an effect abrogated by clorgyline [106]. MYCN is the pivotal neuroblastoma oncogene that is overexpressed in nearly all advanced cases of this neoplasm. Uhl et al. amplified the MYCN oncogene in two neuroblastoma cell lines and evaluated the effect of harmine, a tricyclic β-carboline alkaloid isolated from the harmal plant that acts as a MAO-A inhibitor. Harmine treatment resulted in dose- and time-dependent caspase-mediated apoptosis in MYCN non-amplified but especially in MYCN-amplified neuroblastoma cells. However, mitogen-activated protein kinase and dual-specificity tyrosine phosphorylation-regulated kinase family proteins are also inhibited by harmine so the pro-apoptotic effect might not be exclusively dependent on MAO-A [107].

### Glioma

Glioma represents the most frequently encountered primary brain tumor in humans. Temozolomide (TMZ) is the first-line chemotherapeutic drug used to treat this neoplasm but its efficacy is limited, partly due to the concomitant damage of tumoral and healthy adjacent cells. In the context of a previously described increase of MAO-B activity in human gliomas versus non-tumoral tissues [108], Sharpe et al. demonstrated that MAO-B registers a more than fourfold upregulation in glioma cells, an observation which the authors exploited to develop MP-MUS, a novel prodrug selectively activated by MAO-B. MP-MUS was up to three times more potent than temozolomide in disrupting glioma cells’ viability while simultaneously displaying growth-promoting effects in normal human astrocytes. As it accumulates inside glioma mitochondria, the toxic effect of MP-MUS manifests exclusively toward neoplastic cells [109]. In a subsequent study, the high MAO-B expression demonstrated a positive correlation with the tumor grade, the transcription factor Sp3 and HIF1α, the latter being localized in the nuclei in advanced gliomas or in the cytosol in low-grade gliomas [110]. Since the contribution of MAO-B to glioblastoma is apparent, novel potent and selective MAO-B inhibitors were developed, two examples being Cmp3 and Cmp5. These compounds promote mitochondrial membrane potential depolarization with consequent ROS overproduction in conjunction with Cmp3/5-stimulated downregulation of inducible nitric oxide synthase 2. The resulting oxidative stress decreases glioblastoma cell viability by blocking cell cycle progression in the G1 or G2/M phase. Cmp5 also reduces the expression of MMP-2 and MMP-9, thus mitigating glioma cell migration [111].

A large case–control study indicated that MAO-A polymorphisms might contribute to creating a favorable environment for glioma development. The rs144551722 (single nucleotide polymorphism) SNP of the MAO-A gene was identified as a significant predictor of glioblastoma formation in males, but not in female patients [112].

Gliomas frequently become resistant to temozolomide (TMZ); therefore, the identification of therapies able to destroy cells that have lost their sensitivity toward this drug is paramount. Human TMZ-sensitive and -resistant cell lines express MAO-A, as opposed to normal human astrocytes. NMI, a clorgyline-conjugated near-infrared dye specifically targets tumoral mitochondria and reduces the viability of both TMZ-responsive and -nonresponsive glioma cells. This effect is present as monotherapy and it is enhanced by co-administration of TMZ. Clorgyline and NMI alone or combined with low-dose TMZ improved animal survival in the intracranial TMZ-resistant glioma model with decreased tumor growth and tumor cell proliferation and invasion, reduced micro-vessel density, and increased macrophage infiltration [15]. Since MAO-A and heat shock protein 90 (HSP90) inhibitors have proven their effectiveness against glioblastoma progression, a recent study reported the development of MAO-A/HSP90 dual inhibitors based on isopropylresorcinol and clorgyline. These compounds decreased HER-2 and phospho-Akt cellular expression and suppressed the proliferation of TMZ-sensitive and TMZ-resistant glioblastoma cells while also promoting the decline of tumor growth in a mouse glioblastoma model. MAO-A/HSP90 dual inhibitors have the potential to reduce tumor immune evasion and act as immune checkpoint inhibitors as they limited IFN-γ-induced PD-L1 expression in glioblastoma cells. Programmed cell death protein 1-ligand 1 (PD-L1) is expressed by malignant cells and it further binds to the PD1 receptor present on the surface of activated T cells causing their anergy with consequent tumor immune escape [113].

The natural Chinese herbal remedy plant antimicrobial solution (PAMs) has also been evaluated in the context of glioblastoma. In TMZ-sensitive and TMZ-resistant human glioma cells, PAMs inhibited MAO-A catalytic activity and decreased glioma cell growth, colony formation, and cell migration, while in an in vivo mouse glioblastoma model, this treatment limited tumor growth and increased the survival rate. The beneficial effects were more pronounced after combined administration with low-dose TMZ but were present in PAMs stand-alone therapy as well [114].

### Hematological malignancies

MAO-A registers an increased activity in the cerebrospinal fluid of children diagnosed with acute lymphoblastic leukemia, making the resulting oxidative stress an important contributor to the loss of blood–brain barrier function in these patients [115]. The Reed-Sternberg cells of classical Hodgkin lymphomas highly express MAO-A, especially in the Epstein-Barr virus-negative cases as opposed to primary mediastinal large B-cell lymphomas and mediastinal gray zone lymphoma that display low levels of this enzyme. Nodular lymphocyte-predominant Hodgkin lymphomas, non-Hodgkin lymphomas, and non-neoplastic lymphoid tissues do not seem to exhibit MAO-A expression. Genetic and pharmacological inhibition of MAO-A reduced the growth of human Hodgkin lymphoma cells, while MAO-A overexpression had the opposite effect. Co-administration of clorgyline with the ABVD chemotherapy regimen proved more effective in limiting cell proliferation than either treatment administered as monotherapy. The high MAO-A expression of classical Hodgkin lymphoma might reflect the distinct biology of this malignant condition [116].

### Adrenal cortical carcinoma

Adrenal cortical (AC) carcinomas are rare and generally have a poor prognosis. MAO expression varies in adrenal cortical tissues in accordance to the degree of malignancy. Thus, high expressions of MAO-A and -B were found in tumor cells in AC neoplasm, while MAO-A in stromal cells was more expressed in AC adenoma. Low stromal MAO-A levels in AC neoplasm were associated with a shorter overall survival [117].

### Immune checkpoint role of MAO-A

Along with its effects in cancer cells, MAO-A also influences the immune cell expression of immunosuppressive molecules, known as immune checkpoints [19]. Cancer progression and treatment resistance are promoted by the immunosuppressive properties of the tumor microenvironment, which can be overcome in select cases by the recently developed immune checkpoint blockade therapy comprising the suppression of cytotoxic T lymphocyte–associated protein 4 (CTLA-4) and of the PD1/PD-L1 inhibitory pathway. Despite the initial efficacy of these treatments, tumor recurrence is frequent, most likely due to the existence of multiple immune checkpoint pathways with different roles in accordance to cancer type and disease stages. The identification of new immune checkpoints and the development of targeted therapies against them represent the future of cancer immunotherapy research [25].

Tumor-infiltrating CD8^+^ T cells display the induction of the MAO-A gene, and the high subsequent MAO-A expression is directly correlated with a tolerant phenotype with low responsivity of the immune cells toward tumor antigens, most likely via modified T cell autocrine serotonin signaling. MAO-A knockout or pharmacological inhibition resulted in improved antitumor T cell immunity and suppressed tumor growth in human xenograft or preclinical mouse syngeneic tumor models. Co-administration of MAO inhibitors with anti-PD1 treatments produced synergistic tumor-suppressing effects. Computational clinical data correlation studies revealed a strong connection between intra-tumoral MAO-A expression, T cell dysfunction, and poor patient survival [25]. Tumor-associated macrophages (TAM) play an equally important role in cancer immune evasion. MAO-A induction was reported in mouse and human TAMs where it induces TAM immunosuppressive polarization and depressed anti-tumoral immunity via intensified oxidative stress. MAO inhibition mitigated tumor progression in human xenograft and preclinical mouse syngeneic tumor models, respectively. Co-administration of anti-PD1 treatments had a synergistic tumor-suppressing effect [118].

These results are very promising and support the use of MAO inhibitors to improve the outcome of cancer immunotherapy. However, the required immunotherapeutic doses might induce aggressive behavioral side effects. To avoid MAO inhibitor-associated neurological adverse events while retaining antitumor efficacy, Brown et al. developed crosslinked multilamellar liposomal vesicles based on phenelzine which proved an increased antitumor efficacy as compared to free phenelzine in a mouse melanoma model in the absence of MAO inhibitor-related aggressive behavior [119]. Recently, another novel compound showed great prospects in cancer therapy. A conjugate based on doxorubicin and the MAO-A inhibitor isoniazid (DOX-INH) stimulated CD8^+^ T cell activity in vivo and exhibited a synergistic antitumor effect with a PD-L1 inhibitor. Moreover, in a murine model of orthotopic 4T1 breast cancer, DOX-INH suppressed Shh, IL-6, and TGF-β signaling pathways and thus mitigated EMT and the growth and metastasis of breast cancer cells [120].

As previously discussed, the involvement of MAO in prostate cancer is well documented. Lapierre et al. used the double-knockout MAO-A/PTEN murine prostate adenocarcinoma model to reveal that these mice display an increment in prostatic markers of immune stimulation such as CD8^+^ T cells, granzyme B, and IFN-γ. Concomitantly, prostatic markers of immune inhibition registered a low expression as compared to the PTEN knockout models in which there was no manipulation of the MAO-A gene. These data suggest that antitumor immunity in prostate cancer was amplified by MAO-A deletion and inhibition of this enzyme may alleviate immune suppression in the tumor microenvironment of prostate adenocarcinoma [121].

The various cancer types that overexpress MAO and protective effects of enzyme-modulating interventions are summarized in Table 1.

**Table 1:** Summary of literature data regarding MAO-overexpressing cancer types and related interventions

Tissue type	Experimental model	Main findings	Reference
Prostate	• FFPE human PCa tissues of different Gleason grades • Human PC-3 and LNCaP cell lines	• MAO-A promotes EMT via VEGF-A/NRP1–mediated signaling and consequent activation of AKT/FOXO1-dependent pathways with enhanced nuclear TWIST1 expression • MAO-A regulates HIF1α stability via ROS generation and PHD destruction • MAO-A supports tumor growth and metastasis	[33]
Prostate	• Human PCa tissue microarrays • Human ARCaP_M_, C4-2, PC-3 cell lines, their metastasis-derived sublines	• MAO-A mediates communication between PCa tumor cells, stromal cells, osteoblasts and osteoclasts, therefore facilitating bone and visceral organs PCa metastasis via TWIST1/Shh-IL6-RANKL signaling in the tumor microenvironment	[42]
Prostate	• Human VCaP cell line	• Clorgyline slows VCaP cells proliferation and promotes their secretory differentiation via downregulation of Src, beta-catenin and MAPK oncogenic pathways, involved in androgen-independent growth and metastasis • Clorgyline inhibits tumor growth in vivo	[40]
Prostate	• Human PCa tissue microarrays • Human PC-3, C4-2, DU145, ARCaP_M_ cell lines • Human PrSC and WPMY-1 stromal fibroblast cell lines	• MAO-A promotes ROS production by stromal cells with paracrine TWIST1/IL-6/STAT3 signaling activation and increased tumor invasiveness, stemness, and metastasis potential • Clorgyline inhibits tumor growth, disrupts stromal-cancer cell signaling, and represses cancer stem cell properties by decreasing CD44 expression	[66]
Prostate	• Prostate-specific Pten/MAO-A knockout mouse model • Human 22RV1 and LNCaP cell lines	• MAO-A promotes cancer cell growth, proliferation, and invasiveness, cancer stem cell occurrence, and spheroid formation ability of PCa cells	[35]
Prostate	• Human PCa tissue samples • Human PC-3, LNCaP, 22Rv1, ARCaP_M_ cell lines • Rat PC-12 (neuronal-like) and 50B11 (dorsal root ganglion sensory neuronal) cell lines	• MAO-A is associated with Twist1/SEMA3C/PlexinA2/NRP1 signaling with cMET paracrine or autocrine activation resulting in PCa cell perineural invasion and infiltration • Clorgyline reduces perineural cancer cell invasion along with tumor-infiltrating nerve fiber density, tumor growth, and progression	[43]
Prostate	• Human E-CA-88 and E-CA-90 cell lines derived from Gleason grades 3 and 4 PCa	• Clorgyline seems to reverse the oncogenic transcriptional pathways of beta-catenin and ERBB2 by modifying the gene expression profile regulated by these pathways • Clorgyline has pro-differentiation effects by increasing the expression of the AR and other secretory epithelial cell markers and downregulating the differentiation repressor EZH2	[39]
Prostate	• Human prostatic E-PZ (epithelial) and F-PZ (stromal) cells	• MAO-A prevents basal epithelial cells from differentiating into secretory cells • Clorgyline enhanced the expression of the AR and promoted differentiation of basal epithelial into secretory cells in vitro	[38]
Prostate	• Human LNCaP, PC3, DU145 and C4-2B cell lines • PTEN-deficient PCa mouse model ± survivin KO	• MAO-A expression is enhanced in a survivin-dependent manner during PCa growth • Clorgyline combined with survivin inhibitors synergistically suppresses PCa cell growth, migration, and invasion capacity	[41]
Prostate	• Human PCa tissue microarrays • MAOA/B-KO mouse model • Human normal prostate RWPE-1 cell line	• MAO-A/B ablation causes prostate atrophy with reduced epithelial cell proliferation and decreases basal and progenitor prostate cells • MAO-A/B knockdown and pharmacological inhibition impaired the sphere-forming capacity of prostate stem cells and reduced the CD133^+^/CD44^+^/CD24^−^ stem cell population • Human prostate hyperplasia samples display elevated levels of MAO-A/B	[31]
Prostate	• Human PC3 cell line • Human CAFs obtained from Gleason 4 + 5 PCa tissues	• MAO-A mediated CAF-induced EMT, ROS production, and pro-inflammatory responses (CXCR4 and IL-6 receptor induction) in PC3 cells via mTOR/HIF1α-dependent signaling • Curcumin inhibits MAO-A/mTOR/HIF1α signaling	[58]
Prostate	• Human PCa tissue samples of Gleason grade ≥ 4 + 3 • Human LNCaP and PC3 cell lines	• Docetaxel chemotherapy upregulates MAO-A expression with enhanced PCa cell survival, increased ROS generation and HIF1α expression and nuclear translocation • Clorgyline reduces PCa cell viability after docetaxel chemotherapy	[57]
Prostate	• Human PCa tissue microarrays • Human PC-3, C4-2, DU145, C4-2B^ENZR^ cell lines, normal (PNF1-3) and cancer (PCF1-3) associated primary fibroblast cell lines	• MAO-B expression is upregulated in PCa stroma and promotes PCa cell proliferation, invasiveness, and supports tumor growth • MAO-B induces ROS generation with stromal reactivity and TWIST1/TGFβ signaling with CXCL12/CXCR4 paracrine axis activation that promotes stromal-epithelial interactions and aggressive tumor behavior • Selegiline disrupts stromal-tumor communication and restricts tumor growth	[65]
Prostate	• Human PC-3 and LNCaP cell lines	• Several heptamethine dye–INH conjugates show potent anticancer activity, comparable to that of doxorubicin, while also displaying moderate MAO-A inhibitory activity	[97]
Prostate	• Human LNCaP, DU145, PC-3 cell lines	• Several synthesized flavonoid derivatives (KKRs) non-toxically mitigate LNCaP cells proliferation and DU145 cells migration, respectively, via MAO-A inhibition	[59]
Prostate	• Human LNCaP-LN3 cell line	• As compared to tranylcypromine, pargyline had a greater inhibitory effect on PCa cell proliferation with G1 phase cell cycle arrest, associated with increased apoptotic rate	[64]
Prostate	• Human LNCaP, PC3, DU145 cell lines	• Androgen deprivation-mediated NED of cancer cells in hormone-refractory PCa is facilitated by REST downregulation, leading to MAO-A upregulation • MAO-A-derived ROS inhibit apoptosis and activate autophagy and mitophagy in PCa cells • Pargyline and phenelzine decreased the NED and autophagy of PCa cells	[54]
Prostate	• Human DU145, PC-3, HepG2, MCF-7, LO2 cell lines	• Pc-MLB, a MAO-A targeted photosensitizer, shows high specificity toward PCa cells and inhibits their migration and metastasis	[44]
Prostate	• Human LNCaP, PC-3, DU145, SH-SY5Y cell lines	• PFBL increases MAO-A expression and activity with consequent ROS generation and PCa cell apoptosis • PFBL reduces tumor growth in mice, alone or together with Paclitaxel, while displaying a good safety profile in healthy animals	[61]
Prostate	• Genotyping study in a population of PCa patients • Human LNCaP, PC-3, PC-3 M, 22Rv1 cell lines	• MAO-B rs6324 A-allele is linked with high initial PSA levels, the rs3027452 A-allele is connected to a higher risk of distal metastasis, while the presence of the rs1799836 G-allele can lead to lymph node metastasis and recurrence and is associated with lower MAO-B expression levels • Overexpression of MAO-B decreases PCa cell proliferation, migration, and invasion • Low MAO-B expression is linked to a poorer prognosis due to higher Gleason scores and metastatic capacity and advanced clinical T stages	[63]

*15-LO* 15-lipoxygenase, *ABVD* adriamycin, bleomycin sulfate, vinblastine sulfate, and dacarbazine, *AKT* alpha serine/threonine-protein kinase, *anti-PD-1* anti-programmed cell death protein 1, *AR* androgen receptor, *ARCaP M* derivatives of the ARCaP cell line, with epithelial and mesenchymal characteristics, *Bcl-2* B-cell leukemia/lymphoma 2 protein, *BCG* bacillus calmette guerin, *C4-2B ENZR* enzalutamide-resistant prostate cancer cell line, *CAF* cancer-associated fibroblasts, *CD133* prominin-1, *CD24* heat stable antigen, *CD44* homing cell adhesion molecule, *cHL* classical Hodgkin lymphomas, *CIS* carcinoma in situ, *c-MET* mesenchymal-epithelial transition factor, *Cmp3* compound 3, *Cmp5* compound 5, *CRC* colorectal cancer, *CXCL12* C-X-C motif chemokine ligand 12, *CXCR4* C-X-C chemokine receptor type 4, *DFS* disease-free survival, *DKO* double-knockout, *DMSO* dimethyl sulfoxide, *DOX-INH* doxorubicin-isoniazid conjugate, *DYRK2* dual-specificity tyrosine-phosphorylation-regulated kinase 2, *EBV* Epstein-Barr virus, *EMT* epithelial-to-mesenchymal transition, *ER* estrogen receptor, *ERBB2* erythroblastic leukemia viral oncogene homologue, *ERK1/2* extracellular signal-regulated kinases, *EZH2* depletion of enhancer zeste homolog 2, *FFPE* formalin-fixed paraffin-embedded, *FOXF2* forkhead box F2, *FOXO1* forkhead box protein O1, *GBM* glioblastoma multiforme, *G10*, *G11* MAOA inhibitor-heptamethine carbocyanine dye conjugates, *GLUT1* glucose transporter 1, *HER-2* human epidermal growth factor receptor 2, *HIF-1α* hypoxia-inducible factor 1, *HK2* hexokinase 2, *HPV-16 E7* human papillomavirus E7 oncoprotein, *HSP90* heat shock protein 90, *IL-13* Interleukin 13, *INH* Isoniazid; *IR* ionizing radiation, *KKRs* synthesized flavonoid derivatives, *KO* knockout, *LKB1* liver kinase B1, *MAO-A* monoamine oxidase-A, *MAO-B* monoamine oxidase-B, *MAO-I* monoamine oxidase inhibitors, *MAPK* mitogen-activated protein kinase, *MMP* matrix metalloproteinases, *MP-MUS* N,N-bis(2-chloroethyl)-2-(1-methyl-1,2,3,6-tetrahydropyridin-4-yl)propenamide, *mRNA* messenger Ribonucleic acid, *mTOR* mammalian target of rapamycin, *NE* norepinephrine, *NED* neuroendocrine differentiation, *NF-κB* nuclear factor-Κb, *NHA* normal human astrocytes, *NMI* near-infrared dye MHI-148 conjugated to clorgyline, *NRP1* neuropilin-1, *NSCLC* non-small cell lung cancer, *PAMs* natural plant antimicrobial solution, *Pc-MLB* MAO-A-directed phthalocyanine photosensitizer, *PFBL* polyphenol-rich fraction of Bergenia ligulata, *PGE-2* prostaglandin E, *PHD* prolyn hydroxylase, *PPAR* peroxisome proliferator-activated receptor, *Pten* phosphatase and tensin homolog, *PTX* paclixatel, *RANKL* receptor activator of nuclear factor kB ligand, *REST* repressor element-1 silencing transcription factor, *RNA* ribonucleic acid, *ROS* reactive oxygen species, *SEMA3C* semaphorin 3C, *Shh/IL-6* sonic hedgehog/Interleukin 6, *Sp3* transcription factor Sp3; Src; Proto-oncogene tyrosine-protein kinase Src; *STAT3* signal transducer and activator of transcription 3, *STS* staurosporine, *TAM* tumor-associated macrophages, *TGFβ* transforming growth factor beta, *TMZ* temozolomide, *TNBC* triple negative breast cancer, *TWIST1* twist-related protein 1, *UC* urothelial carcinoma, *VEGF* vascular endothelial growth factor, *WT* wild-type

**Fig. 1:**
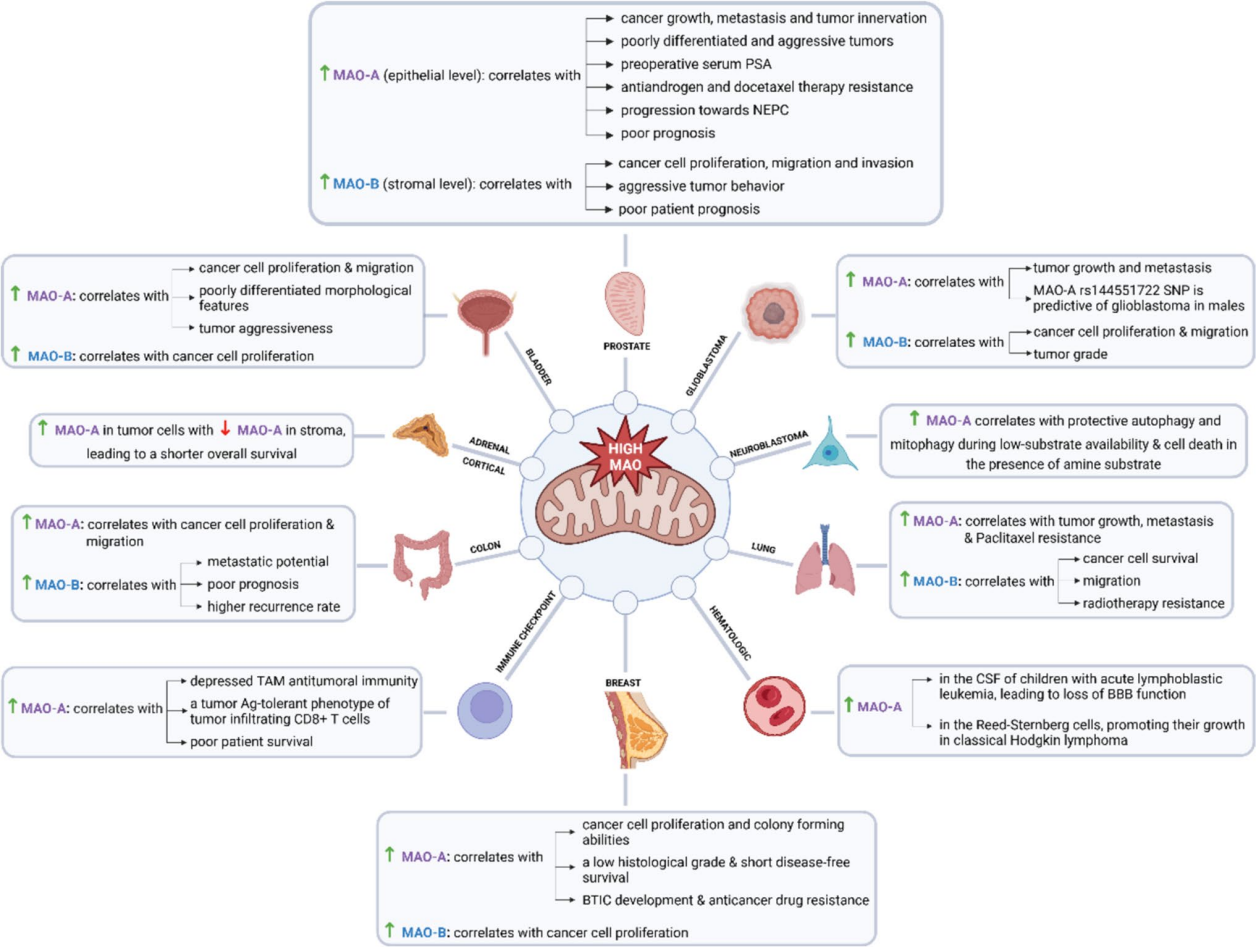
Cancer-promoting effects of MAO upregulation. Ag antigen, BBB blood–brain barrier, BTIC breast tumor-initiating cells, CSF cerebrospinal fluid, NEPC neuroendocrine prostate cancer, PSA prostate-specific antigen, SNP single nucleotide polymorphism, TAM tumor-associated macrophages. (Figure created with a licensed version of BioRender.com.)

Figure 1 illustrates the cancer-promoting effects of MAO upregulation.

## MAO down-regulation in tumors

Studies on the involvement of MAO in cancer have been carried out for more than 60 years. As previously stated, in 1959, Wattenberg described a low MAO activity profile in colon carcinoma cells and connected it to their aggressive traits [13]. A wealth of literature has been accumulating since then in support of MAO downregulation in different tumor tissues. Rybaczyk et al. analyzed information from GEO profiles database and reported a significant downregulation of MAO-A in 95.4% of human cancer patients and 94.2% of animal cases, as opposed to their paired non-cancerous controls. The evaluated pathologies were cutaneous malignant melanoma, lung neuroendocrine tumor, malignant pleural mesothelioma, breast cancer, and hypopharyngeal cancer [122]. However, since MAO expression was measured in complex samples, the possibility that the aforementioned downregulation had occurred in resting and not actively dividing cells cannot be excluded. By comparison with MAO-overexpressing tumors, cancer types that associate a low MAO expression and activity profile are fewer and are mostly gastroenterological in nature.

### Gastro-intestinal neoplasms

Neurotransmitters are of utmost importance for normal and abnormal functions in the digestive system and it seems that their secretion is paramount for peripheral nerves to achieve tumor innervation and take control of the tumor microenvironment [123]. Thus, the role of MAO in gastrointestinal malignancies was explored quite early on. Initial studies showed that MAO activity was significantly decreased in samples of gastric cancer of the antrum or of the body as compared to the surrounding uninvolved mucosa [124]. These results were later confirmed by Ojetti et al. who performed cDNA microarray molecular profiling of gastric cancers with N0 or N + lymph node status and found that the MAO-A gene was exclusively expressed in the N0 group. Moreover, it was downregulated versus the healthy gastric mucosa [125]. More recent research has focused on investigating the role of MAO-A in glycolysis and proliferation of gastric cancer cells. Wang et al. discovered a substantial increase in norepinephrine concentration in gastric cancer tissues, which was correlated with activated glycolysis. This finding is of great significance since the rapid proliferation of tumor cells is heavily reliant on the energy supplied by glycolysis. The accumulation of norepinephrine in the cancer tissue prompted the authors to assess the expression of MAO-A and MAO-B which was found to be low for both isoforms. Their reduced level seems to be correlated with a high expression or activity profile of key genes controlling the glycolysis and interferon-γ pathways. In line with this observation, MAO-A and -B mRNA expression exhibited a negative correlation with the SUVmax value in PET-CT scans [126]. Conversely, in their study population, Chen et al. found a mostly increased gastric cancer MAO-A expression as opposed to paired adjacent benign tissues. Silencing MAO-A expression inhibited malignant cell viability and migratory capacity and modulated the mitochondrial function of cancer cells by decreasing the generation of mitochondrial ROS, enhancing ATP production and blocking aerobic glycolysis. The authors observed that in cancer tissue samples, overexpressing MAO-A the glycolysis-stimulating effect was in conjunction with the high expression of HK2 and the low expression of pyruvate dehydrogenase (PDH) [127]. However, a very recent study observed a tight association between MAO-A downregulation in gastric adenocarcinoma samples and poor patient prognosis. When MAO-A overexpression was induced using in vitro or in vivo experimental models, tumor growth was significantly inhibited. A more in-depth analysis regarding the involved mechanisms revealed that MAO-A has a positive correlation with the anti-oncogenic protein NDRG1. When both are overexpressed, their interaction inhibits the Warburg effect (aerobic glycolysis providing energy to cancer cells) by suppressing the PI3K/Akt/mTOR pathway, thereby restricting cancer progression. Invaluable previous work has shown that in a PI3K/Akt pathway-dependent manner, HK2 is overexpressed and PDH is downregulated in cancer cells, thus supporting the Warburg effect [128]. Treatment of the MAO-A overexpressing cells with clorgyline, the selective irreversible MAO-A inhibitor, abrogated the enzyme-dependent tumor-suppressing effects [129]. Using information from database repositories, Chang et al. applied successive statistical regression analysis techniques and found that the MAO-A gene is one of the four mitochondrial-related parameters that are differentially expressed between normal and stomach adenocarcinoma samples, or between low- and high-risk groups. Lower expression of the MAO-A gene and protein was prevalent in gastric cancer tissue versus the healthy controls, and patients with a higher expression of MAO-A had a shorter overall survival [130]. Although some of the above-mentioned study results seem contradictory, it is clear that MAO expression imbalance in stomach carcinoma deeply influences malignant cell metabolism and impacts the prognosis of the patient. Different microenvironmental conditions can possibly change the interaction pattern of MAO with up- or downstream molecules. Infection of AGS human gastric adenocarcinoma cells with Helicobacter Pylori increases MAO-A expression [131]. Conversely, the Epstein–Barr virus, another etiological factor of gastric cancer, downregulates MAO-A expression in pre-malignant or malignant nasopharyngeal epithelial cells [23], while smoking hinders MAO activity in the central nervous system as well as peripheral tissues [132]. Moreover, depending on their level, mitochondria-generated ROS can display tumor-supporting or tumor-suppressing effects [133]. Patient survival is a parameter that is difficult to accurately assess, especially when using small sample groups, as it is reliant on a myriad of factors that include tumor type, patient’s characteristics, such as age or family history, the degree of systemic inflammation, and nutritional indicators, such as BMI or tumor markers, like CEA [134].

The search for new biomarkers has extended into the field of esophageal cancer as well, where MAO expression seems to be preponderantly low. An earlier study performed on 50 patients revealed that both MAO activity as well as MAO-A expression were decreased in the vast majority of cases [135]. A later study described that esophageal cancer tissues display high levels of dopamine, especially in tumors originating from patients bearing lymphatic metastasis. This occurrence was linked to a decrease in the expression of MAO-A and MAO-B, and overexpression of the dopamine-generating tyrosine hydroxylase. Activation of the upregulated D5 dopamine receptor promotes the proliferation and inhibits the apoptosis of esophageal cancer cells via the Akt/mTOR pathway with enhanced expression of c-MYC and HIF1α and stimulation of the Warburg effect [136]. Recently, by mining the TCGA dataset for differential expressed genes (DEG) in esophageal cancer and overlapping them with mitochondria-related genes, 306 mitochondria-related DEGs were established. The statistical processing of the resulting data revealed the MAO-B gene as a prognostic predictor for patients with esophageal cancer. Its downregulation was confirmed by further analysis, while higher expression of MAO-B was linked to an improved overall survival [137].

### Accessory digestive organs neoplasms

In cholangiocarcinoma cells, Huang et al. demonstrated that coordinated epigenetic and IL-6-dependent events are responsible for the suppressed expression of MAO-A. Patients displaying this phenotype had enhanced cancer invasiveness associated with poor survival rates. Conversely, restoration of MAO-A had growth-inhibiting effects [138]. These results are in agreement with other data confirming a decreased MAO-A mRNA expression in cholangiocarcinoma that leads to serotonin accumulation at this level. Moreover, supplementary serotonin administration supported malignant cell growth, whereas the inhibition of its synthesis inhibited tumor development [139].

Similar effects were reported in human and murine pancreatic adenocarcinoma tissue where the high levels of serotonin were ascribed to the local deregulation of serotonin-controlling enzymes. Tryptophan hydroxylase 1 (TPH1), the enzyme responsible for peripheral serotonin synthesis, was upregulated, while MAO-A expression was significantly lower than that in the normal pancreas. These changes were correlated with the size and stage of the tumor mass and a shorter overall patient survival. The detrimental effects of serotonin were mediated by the 5-hydroxytryptamine receptor 2B (HTR2B) receptors. Upon activation, they promoted cancer cell proliferation and prevented apoptosis via PI3K/Akt/mTOR signaling with increased protein levels of MYC and HIF1α and stimulation of the Warburg effect [140].

Serotonin is not the only neurotransmitter whose accumulation has cancer-promoting effects. Li et al. described a significant downregulation of MAO-A in hepatocellular carcinoma (HCC), most likely due to epigenetic methylation and histone acetylation. Consequently, the authors noted a buildup of norepinephrine and epinephrine which promoted metastasis and prevented anoikis, effects which were abrogated by inducing MAO-A overexpression [22]. In line with these observations, Yan et al. noted that in HCC, the dopamine-generating enzyme dopa decarboxylase was upregulated, while MAO-A was downregulated, leading to a dopamine imbalance that promoted HCC proliferation and metastasis via the DRD1 dopamine receptor [141]. Noteworthy, a recent study evaluating the effects of antidepressants in HCC cells described that the administration of moclobemide, the reversible MAO-A inhibitor, did not decrease HCC cell viability [142], supporting previous reports of a low MAO-A expression in this type of cancer tissue.

### Otorhinolaryngologic neoplasms

Recently, Qi et al. stated that in head and neck squamous cell carcinoma (HNSCC), MAO-B is significantly downregulated, while its experimentally induced overexpression promotes the apoptosis and inhibits the proliferation, migration, and invasion of the malignant cells. These effects were reversed by selegiline [143]. Nasopharyngeal carcinoma is a particular subtype of HNSCC that has been consistently associated with the Epstein-Barr virus (EBV) infection [144]. It seems that in both pre-malignant as well as malignant nasopharyngeal epithelial cells, EBV downregulates MAO-A expression, partly via IL-6/STAT3-mediated signaling and epigenetic silencing through methylation of the MAO-A gene promoter. In turn, the low MAO-A expression facilitates nasopharyngeal carcinoma cell migration [23]. A subsequent study performed in oral and pharyngeal cancer tissues by Chen et al. simultaneously evaluated MAO-A and MAO-B mRNA and protein expression at this level and found them to be markedly decreased as compared to the adjacent non-tumoral sites. Moreover, the risk of oral cavity and pharynx cancers was much higher in carriers of the MAOA rs6323 G-allele and MAOB rs6324 G-allele [145]. An in silico study aimed at revealing novel therapeutic approaches in oral squamous cell carcinoma described reduced expression of MAO-B in this cancer type. Therefore, the performed molecular docking experiments were designed to identify drugs that could inhibit the tumor-inducing overexpression of Akt1 and Akt2 without simultaneous inhibition of MAO-B [146]. A particularity of cancers arising in the oral cavity is their constant contact with an easily obtainable biological sample, namely saliva. Salivary MAO-B mRNA levels were demonstrated to be significantly low in a study population of oral squamous cell carcinoma patients under 60 years of age [147]. This is of great importance since the possibility of cancer biomarker assessment via non-invasive procedures holds great potential as a sensitive screening tool and as a means to expedite cancer diagnosis.

### Cancers with poorly explored MAO expression

microRNAs (miR) have garnered considerable research attention as regulatory factors in oncogenesis and potential anticancer therapeutic targets but information regarding a possible interaction between MAO and these molecules is scarce. In endometrial carcinoma, miR-522 was found to be highly expressed leading to MAO-B downregulation which promoted cell proliferation and migration. Co-transfection of an overexpression vector of MAO-B along with miR-522 mimics into endometrial carcinoma cells suppressed their proliferative and migratory capacity, demonstrating that the tumor-promoting effects of miR-522 are mediated by MAO-B expression downregulation [148].

Benign tumors can exhibit modified MAO expression patterns as well. In pheochromocytoma, the tumoral protein level of MAO-A and -B was significantly lower than that of healthy adrenal medulla tissue, while mRNA for MAO-A, but not MAO-B, followed the same decreasing trend. As a result, the increased substrates (epinephrine and norepinephrine) availability for catechol-O-methyltransferase translates into overproduction of metanephrines. This is consistent with the current use of plasma and urine metanephrines as diagnostic markers for pheochromocytoma [149].

The literature data reporting the MAO downregulation in various cancer types are summarized in Table 2.

**Table 2:** Summary of literature data regarding cancer types associated with MAO downregulation

Tissue type	Experimental model	Main findings	Reference
Gastric cancer	• Human gastric cancer tissue samples	• MAO activity is significantly decreased in gastric cancer samples extracted from the antrum/body as compared to the surrounding uninvolved mucosa	[124]
Gastric cancer	• Human gastric cancer tissue samples	• The MAO-A gene is exclusively expressed in the N0 (node involvement negative) group in which MAO-A is downregulated	[125]
Gastric cancer	• Human gastric cancer tissue samples • Human BGC-823, HGC-27 cell lines • In silico biostatistical analysis (TCGA database interrogation)	• MAO-A and MAO-B expression is decreased in gastric cancer tissue, leading to norepinephrine accumulation which further promotes tumor cell glycolysis • MAO-A and MAO-B mRNA expression is negatively correlated with the PET-CT SUVmax value and the glycolysis and interferon γ pathways, respectively • Although not reaching statistical significance, low MAO-A expression associates with a larger ratio of PD-L1 positive samples	[126]
Gastric cancer	• Human gastric cancer tissue samples • Human NCI-N87 MGC803, AGS cell lines	• MAO-A is significantly upregulated in gastric cancer tissues and cell lines and represents a positive regulator of gastric cancer cell proliferation and migration • Silencing MAO-A expression suppresses migration and invasion of gastric cancer cells, with decreased ROS and increased ATP generation along with aerobic glycolysis inhibition in the studied cell lines	[127]
Gastric cancer	• Human and mouse gastric cancer tissue samples • Human BGC-823, HGC-27, SGC-7901, MGC-803, MKN-45 and AGS cell lines • In silico biostatistical analysis (TCGA and GEO database interrogation)	• MAO-A is downregulated in gastric cancer and correlates with a poor prognostic • MAO-A attenuates glycolysis and inhibits gastric cancer progression via PI3K/Akt/mTOR pathway inhibition	[129]
Gastric cancer	• Human SGC-7901, HGC-27 cell lines • In silico biostatistical analysis (TCGA and GEO database interrogation)	• MAO-A is expressed at lower levels in gastric cancer tissues and it was included in a mitochondrial-related risk prognostic model that can be employed to personalize the treatment of gastric cancer patients according to their tumor characteristics	[130]
Esophageal cancer	• Human primary esophageal cancer tissue samples	• MAO expression and activity are decreased in esophageal cancer tissues	[135]
Esophageal cancer	• Human esophageal cancer tissue samples • Human HEEC, ECA109, EC9706, KYSE30, TE-1, and KYSE140 cell lines	• Dopamine accumulates in esophageal cancer tissues, partly due to MAO-A and -B downregulation • Dopamine acting on its upregulated D5 receptor promotes cancer cell proliferation along with the Warburg effect and inhibits the apoptosis of cancer cells	[136]
Cholangiocarcinoma	• Human hilar cholangiocarcinoma tissue samples • Human Mz-ChA-1 cell line	• MAO-A mRNA expression is significantly decreased in the malignant samples via promoter hypermethylation and IL-6 signaling • Induced MAO-A overexpression mitigates tumor growth and invasion	[138]
Cholangiocarcinoma	• Commercially available cholangiocarcinoma tissue arrays • Human Mz-ChA-1, HuH-28, HuCC-T1, CCLP1, SG231, TFK-1 cell lines	• MAO-A expression is reduced, while local serotonin secretion is increased in cholangiocarcinoma cells/tissues • The low MAO-A expression was not correlated to the degree of tumor differentiation • The increased local serotonin concentration promotes cholangiocarcinoma cell growth	[139]
Pancreas	• Human PDAC tissue samples • Kras^G12D/+^/Trp53^R172H/+^/Pdx1-Cre (KPC) mouse model for PDAC • Human PDAC AsPC-1, BxPC-3, Capan-2, CFPAC-1, SW1990, HPAC, PANC-1 cell lines • In silico biostatistical analysis (Oncomine and TCGA database interrogation)	• The decreased MAO-A expression in PDAC cells and tissues negatively correlates with tumor stage and size and predicts a shorter patient survival time • PDAC displays high serotonin levels which promote the proliferation and inhibit the apoptosis of cancer cells via the HTR2B • Serotonin supports the growth of pancreatic tumors and activates the PI3K–Akt–mTOR pathway along with the Warburg effect via increased HIF1α and MYC protein levels	[140]
Hepatocellular carcinoma	• Human HCC tissue samples • Human MHCC97‐H, MHCC97‐L, SK‐HEP‐1, PLC/PRF/5, Huh‐7, Hep‐3B, Hep‐G2 cell lines	• In HCC, local accumulation of dopamine occurs, partly due to MAO-A downregulation • Dopamine facilitates HCC cell proliferation, migration, and invasion • The upregulated dopamine receptor DRD1 is linked to a poor prognosis due to the activation of the cAMP/PI3K/AKT/CREB pathway, leading to HCC cell proliferation and metastasis	[141]
HNSCC	• Human HNSCC tissue samples • In silico biostatistical analysis (TCGA database interrogation) • Human HSC3, Cal27, HN4, HN6, and Fadu cell lines	• HNSCC tissues and cell lines express low MAO-B levels • Induced MAO-B overexpression promotes HNSCC cell apoptosis and suppresses proliferation, migration, and invasion via the inhibition of the MAPK pathway • Selegiline partially reverses MAO-B overexpression-induced phenotypes	[143]
Nasopharyngeal carcinoma	• Human NPC tissue samples • Human EBV^−^ (NE1, CNE2, HK1, HONE1, SUNE1, TW01, TW04) and EBV^+^ (C666-1) cell lines	• MAO-A is downregulated in NPC, which supports cancer cell migration • EBV infection reduces MAO-A levels both in pre-malignant as well as malignant nasopharyngeal epithelial cells • MAO-A downregulation occurs via IL-6/IL-6R/STAT3 signaling and epigenetic silencing, effects which might be induced by the EBV infection	[23]
Oral and pharyngeal cancers	• Human oral and pharyngeal cancer tissue samples • Human OECM-1 and HSC-3 cell lines	• The MAO-A rs6323 G-allele and MAO-B rs6324 G-allele increase the risk of oral and pharyngeal cancers • MAO-A and MAO-B mRNA levels were significantly decreased in oral and pharyngeal cancerous tissues • MAO-A and MAO-B mRNA levels can represent biomarkers that differentiate tumoral from normal tissue	[145]
Oral squamous cell carcinoma	• In silico drug design and molecular docking studies	• Downregulation of MAO-B is associated with the development of OSCC • The cytochrome P450 inhibitors Galuteolin and Linarin are potential therapeutic leads for OSCC, by targeting Akt1 and Akt2 without affecting MAO-B expression	[146]
Oral squamous cell carcinoma	• Human saliva samples • In silico biostatistical analysis (GEO database interrogation)	• MAO-B expression is downregulated in the OSCC group • The combined assessment of MAO-B and NAB2 proved to be highly predictive of OSCC, particularly in the under 60 years age group	[147]
Endometrial carcinoma	• In silico biostatistical analysis (TCGA database interrogation) • Human HEC-1A, KLE, HHUA cell lines	• Increased levels of miR-522 in endometrial carcinoma downregulate MAO-B expression and are associated with cancer cell proliferation, migration, and invasion, leading to a poor prognosis • Upregulation of MAO-B attenuated the cancer-promoting effects of miR-522	[148]
Pheochromocytoma	• Human pheochromocytoma tissue samples	• MAO-A is consistently reduced in pheochromocytoma tissue, while MAO-B is downregulated at the protein level despite no significant change in mRNA levels • Low MAO-A expression in the pheochromocytoma tissue results in diminished oxidation of catecholamines	[149]

*5-HT* serotonin, *DRD1* dopamine receptor D1, *HCC* hepatocellular carcinoma, *HNSCC* head and neck squamous cell carcinoma, *HTR2B* 5-hydroxytryptamine receptor 2B, *NAB2* NGFI-A binding protein 2**,** *NPC* nasopharyngeal carcinoma, *OSCC* oral squamous cell carcinoma, *PDAC* pancreatic ductal adenocarcinoma, *PD-L1* programmed death-ligand 1, *PET-CT* positron emission tomography, *SUVmax* maximum standardized uptake values

**Fig. 2:**
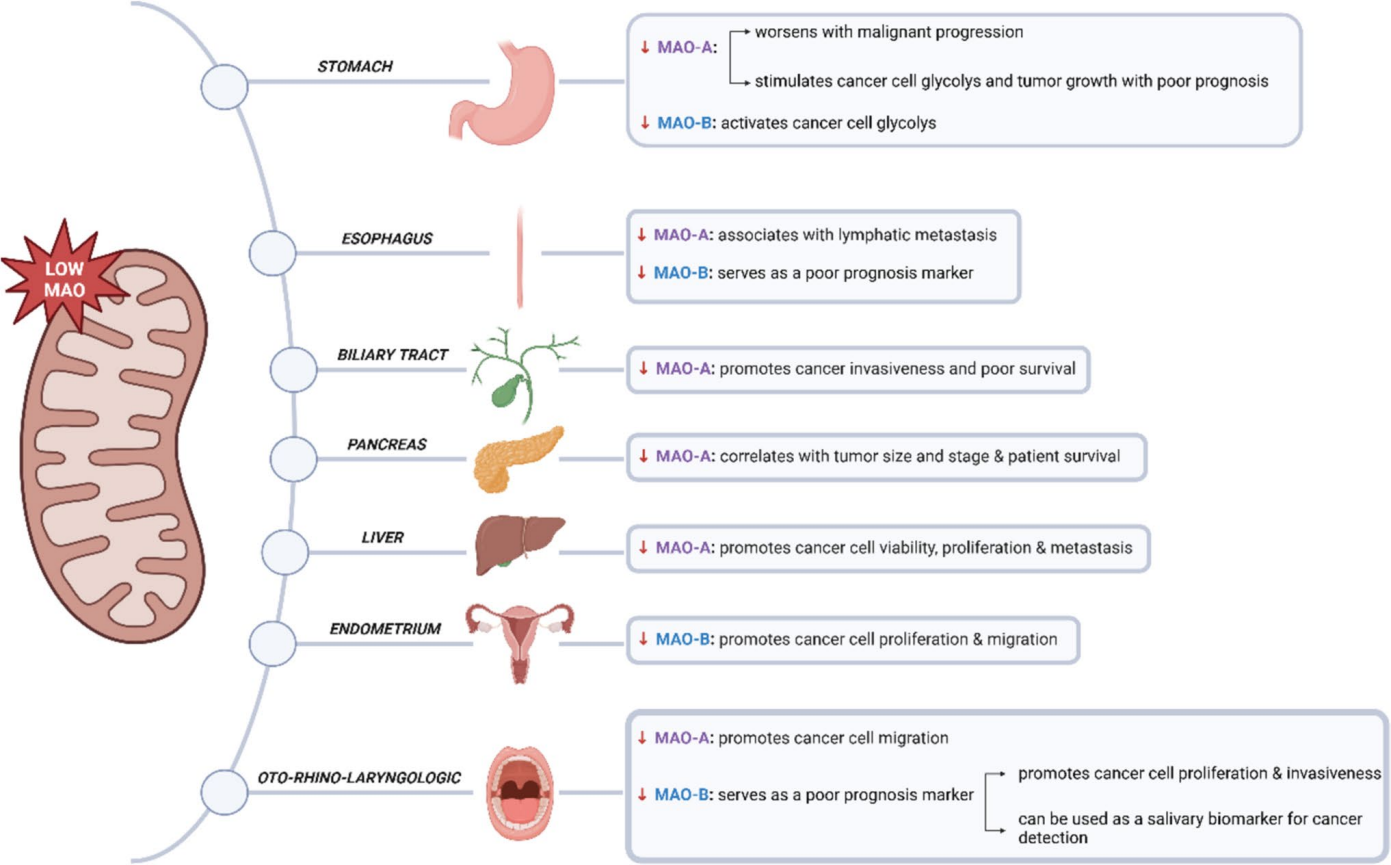
Cancer-promoting effects of MAO downregulation. (Figure created with a licensed version of BioRender.com)

Figure 2 illustrates the cancer-promoting effects of MAO downregulation.

## MAO expression or activity assessment as a possible novel cancer biomarker

It is estimated that approximately 50% of cancers are detected in an advanced stage, thus limiting treatment options and decreasing survival rates [150]. The early and non-invasive detection of biological changes that differentiate normal from tumoral tissue represents a pivotal goal for cancer research. A growing body of evidence has revealed that MAO holds tremendous potential as a diagnostic biomarker and the assessment of its expression or enzyme activity in case of suspicion of certain malignancies could identify individuals at risk who should be closely monitored for early cancer detection or for prognosis evaluation [22, 122, 138, 143]. Although targeted radiotracers for MAO imaging through in vivo positron emission tomography (PET) scans had been intensely studied for neurological pathologies, two decades ago, Fowler et al. revealed PET scan mapping of MAO-A and -B activity in peripheral organs in humans to be a feasible enzyme quantification method [151–153]. Consequently, a MAO-A reversibly binding radiotracer showed great promise in autoradiography studies in detecting the high enzyme expression in human bladder cancer and neuroendocrine gastro-entero-pancreatic tumors [154, 155]. Recently, in a LNCaP tumor-bearing animal model, this tracer was proven an attractive PET probe that confirmed the vastly described overexpression of MAO-A in aggressive prostate cancer [156]. In order to further enhance convenience of use and cost-effectiveness, fluorescent probes were next introduced for the detection of MAO and soon revealed satisfactory results [157]. NMI, a near-infrared dye conjugated with the MAO-A inhibitor clorgyline, specifically targeted tumor tissue in a mouse xenograft model of prostate cancer, without enrichment in normal areas. Moreover, it actively inhibited malignant cell proliferation, colony formation, migration, and invasion, revealing both a therapeutic and diagnostic potential [158]. The positive results of NMI biodistribution kinetics were recapitulated in a glioma mouse model [159], while in an intracranial mouse xenograft model of temozolomide-resistant glioma, NMI had a tumor-specific localization, mitigated tumor growth, and promoted animal survival [15]. More recently, in a murine patient-derived xenograft model of colorectal cancer with MAO-A overexpression, NMI significantly reduced tumor volume even when the administered dose was 10 times lower than that of clorgyline without displaying toxicity [160]. Using NCI60 screening, Feng et al. reviewed the 60 human cell lines from the available 9 types of cancer and found that in various MAO-overexpressing lines of central nervous system, prostate and non-small cell lung cancers NMI inhibited cancer cell growth with an in vitro efficacy that outperformed existing chemotherapeutic agents. However, given the lack of cell-tumor microenvironment interaction, these results must be recapitulated in vivo before NMI can be established as a “theranostic” anticancer drug [161]. In a murine prostate cancer xenograft model, another near-infrared fluorescent dye conjugated with the MAO-A inhibitor isoniazid exhibited specific targeting of the tumor tissue and suppressed its growth, with good safety and no obvious treatment toxicity, as opposed to traditional chemotherapy [162]. A two-photon fluorescent probe based on moclobemide also efficiently and selectively mapped prostate cancer cells and suppressed their growth and metastasis potential [163]. Research using MAO inhibitor-based small molecule PET probes has focused on cancers overexpressing this enzyme. However, as previously stated, several tumor types in different organs are associated with MAO downregulation. Provided that future studies will validate MAO expression as a diagnostic or prognostic tumor marker, PET scans could identify MAO “hot” or “cold” spots that could be associated with the existence and extent of a malignant process or with patient prognosis. Moreover, pre- to post-therapeutic changes in MAO expression in a certain tumor tissue might be able to evaluate treatment efficacy in clinical practice, supporting the development of personalized medicine.

Besides imaging procedures, other non-invasive investigations have been able to reveal the presence of cancer. More than 4 decades ago, Feldman demonstrated that in patients with various metastasized biopsy-proven malignant neoplasms, platelet MAO activity was significantly higher [164]. Rampling et al. confirmed these results stating that regardless of platelet count, patient age, gender, treatment received or smoking habit, platelet MAO activity was increased across different types of neoplasms except for breast cancer [165]. However, it is important to mention that other confounders such as inflammation might be involved in elevating MAO-B activity [166]. More recently, salivary MAO-B mRNA levels were demonstrated to be significantly low in patients under 60 years of age diagnosed with oral squamous cell carcinoma [147]. Although more studies are necessary to validate this finding in larger populations, salivary MAO-B mRNA could represent a non-invasive biomarker for the early diagnosis of this neoplasm.

## Conclusions and future directions

Over the past decades, cancer has persistently remained among the top causes of death worldwide despite the numerous oncological advances, which are inadvertently associated with an enormous time and money expenditure. In this context, the strategy of drug repurposing seems a promising avenue for future cancer research. A wealth of literature garnered during the past century shows the immense potential that MAO holds in the field of cancer diagnostics and therapeutics. However, there is still much to be uncovered regarding the mechanisms that govern the differential expression of this enzyme in various cancer types. Tumor-specific MAO-dependent signaling pathways must be deciphered in order to devise treatment regimens adapted for each oncological subspecialty. Moreover, population characteristics might be of significance when attempting to extrapolate study conclusions obtained in a specific category of patients to the general population. Still, it is very promising that MAO inhibitors have recently been evaluated in a phase 2 prostate cancer clinical trial where phenelzine proved a beneficial effect and displayed a good safety profile. Larger clinical trials with long-term follow-up will need to be performed in order to assess the efficacy of MAO inhibitors in different cancer types and in conjunction with various established chemotherapeutic agents.

## Supplementary Information

Below is the link to the electronic supplementary material.

Supplementary file1 (PDF 366 KB)

## Data Availability

No datasets were generated or analysed during the current study.

## Abbreviations

ABVD: Adriamycin, bleomycin sulfate, vinblastine sulfate, and dacarbazine
AC: Adrenal cortical
Akt/FOXO1: Alpha serine/threonine-protein kinase/Forkhead box protein
Akt2: AKT serine/threonine kinase 2
AMPK: AMP-activated protein kinase
AMPKα1: AMP-activated protein kinase α1
anti-PD1: Anti-programmed cell death-1 monoclonal antibody
API: Androgen pathway inhibitors
AR: Androgen receptor
AR-V7: Androgen receptor variant 7
BMI: Body mass index
BTIC: Breast tumor-initiating cells
CD44: Cluster of differentiation 44
cDNA: Complementary deoxyribonucleic acid
CEA: Carcinoembryonic antigen
cMET: Mesenchymal-epithelial transition factor
Cmp3: Compound 3
Cmp5: Compound 5
c-Myc: Cellular myelocytomatosis oncogene
CRPC: Castration-resistant prostate cancer
CTC: Circulating tumor cells
CTLA-4: Cytotoxic T lymphocyte–associated protein 4
CXCL12: C-X-C motif chemokine ligand 12
CXCR4: C-X-C chemokine receptor type 4
DEG: Differentially expressed genes
DNA: Deoxyribonucleic acid
DOX-INH: Doxorubicin-isoniazid conjugate
DRD1: Dopamine receptor D1
EBV: Epstein-Barr virus
EMT: Epithelial-to-mesenchymal transition
ER: Estrogen receptor
ERBB2: Erythroblastic leukemia viral oncogene homologue
EZH2: Enhancer of zeste homolog 2
FOXA1: Forkhead box protein A1
GEO: Gene expression omnibus
GLUT1: Glucose transporter 1
GR: Glucocorticoid receptor
GSK-3β: Glycogen synthase kinase 3 beta
HCC: Hepatocellular carcinoma
HER-2: Human epidermal growth factor receptor 2
HIF1α: Hypoxia-inducible factor 1-alpha
HK2: Hexokinase 2
HNSCC: Head and neck squamous cell carcinoma
HPV: Human papillomavirus
HSP90: Heat shock protein 90
HTR2B: 5-Hydroxytryptamine receptor 2B
IFN-γ: Interferon γ
IL-2: Interleukin 2
IL-6: Interleukin 6
MAO-A: Monoamine oxidase-A
MAO-B: Monoamine oxidase-B
MET: Mesenchymal-to-epithelial transition
miR-522: MicroRNA 522
MMP14: Matrix metallopeptidase 14
MMP2: Matrix metallopeptidase 2
MMP-9: Matrix metallopeptidase 9
MPC3: Mitochondrial pyruvate carrier 3
mRNA: Messenger ribonucleic acid
mTOR: Mammalian target of rapamycin
MYCN: Avian myelocytomatosis viral oncogene neuroblastoma derived homolog
NDRG1: N-myc downstream-regulated gene 1 protein
NEPC: Neuroendocrine prostate cancer
NF-κB: Nuclear factor-κB
NMI: Near-infrared MAO-A inhibitor
NMIBC: Non-muscle invasive bladder cancer
NRF2: Nuclear factor erythroid 2–related factor 2
NRP1: Neuropilin-1 receptor
NSCLC: Non-small cell lung cancer
p38MAPK: P38 mitogen-activated protein kinases
p53: Tumor protein p53 gene
PAMs: Plant antimicrobial solution
PBMC: Peripheral blood mononuclear cells
Pc-MLB: MAO-A-directed phthalocyanine photosensitizer
PD-1: Programmed cell death protein 1
PDH: Pyruvate dehydrogenase
PD-L1: Programmed cell death protein 1 Ligand 1
PET-CT: Positron emission tomography-computed tomography scan
PFBL: Polyphenol-rich fraction of Bergenia ligulata
PI3K: Phosphoinositide 3-kinases
PPARγ: Peroxisome proliferator-activated receptor γ
PR: Progesterone receptor
PSA: Prostate-specific antigen
PTEN: Phosphatase and tensin homolog
RANKL: Receptor activator of nuclear factor kB ligand
REST: Repressor element-1 silencing transcription factor
ROS: Reactive oxygen species
SCLC: Small cell lung cancer
Shh: Sonic hedgehog
SNP: Single nucleotide polymorphism
Sp3: Specificity protein 3
SSRI: Selective serotonin reuptake inhibitors
STAT3: Signal transducer and activator of transcription 3
STAT6: Signal transducer and activator of transcription 6
SUVmax: Maximum standardized uptake value
TAM: Tumor-associated macrophages
TCGA: The cancer genome atlas
TGFβ: Transforming growth factor beta
TMZ: Temozolomide
TNBC: Triple-negative breast cancer
TPH1: Tryptophan hydroxylase 1
TWIST1: Twist-related protein 1
VEGF: Vascular endothelial growth factor
VNTR: Variable number of tandem repeat
YAP1: Yes-associated protein 1
